# National guidelines for smoking cessation in primary care: a literature review and evidence analysis

**DOI:** 10.1038/s41533-016-0004-8

**Published:** 2017-01-20

**Authors:** Marjolein Verbiest, Evelyn Brakema, Rianne van der Kleij, Kate Sheals, Georgia Allistone, Siân Williams, Andy McEwen, Niels Chavannes

**Affiliations:** 1 National Institute for Health Innovation, School of Population Health, The University of Auckland, Auckland, New Zealand; 2 Centre for Longitudinal Research - He Ara ki Mua, School of Population Health, The University of Auckland, Auckland, New Zealand; 3 Department of Public Health and Primary Care, Leiden University Medical Centre, Leiden, The Netherlands; 4 Department of Clinical, Educational and Health Psychology, University College London, London, UK; 5 National Centre for Smoking Cessation and Training, London, UK; 6 International Primary Care Respiratory Group (IPCRG), Westhill, Scotland, UK

## Abstract

National guidelines for smoking cessation in primary care can be effective in improving clinical practice. This study assessed which parties are involved in the development of such guidelines worldwide, which national guidelines address primary care, what recommendations are made for primary care settings, and how these recommendations correlate with each other and with current evidence. We identified national guidelines using an online resource. Only the most recent version of a guideline was included. If an English version was not available, we requested a translation or summary of the recommendations from the authors. Two researchers independently extracted data on funding sources, development methodologies, involved parties, and recommendations made within the guidelines. These recommendations were categorised using the pile-sort method. Each recommendation was cross-checked with the latest evidence and was awarded an evidence-rating. We identified 43 guidelines from 39 countries and after exclusion, we analysed 26 guidelines (22 targeting general population, 4 targeted subpopulations). Twelve categories of recommendations for primary care were identified. There was almost universal agreement regarding the need to identify smokers, advice them to quit and offer behavioural and pharmacological quit smoking support. Discrepancies were greatest for specific recommendations regarding behavioural and pharmacological support, which are likely to be due to different interpretations of evidence and/or differences in contextual health environments. Based on these findings, we developed a universal checklist of guideline recommendations as a practice tool for primary care professionals and future guideline developers.

## Smoking cessation support in primary care: universal guidelines sought

An international team call for a universal guideline for primary-care practitioners who help patients to stop smoking. Although many nations have such guidelines, no studies have examined whether these guidelines are consistent with the current evidence. Marjolein Verbiest at the National Institute for Health Innovation, The University of Auckland, New Zealand, and co-workers of the International Primary Care Respiratory Group and the National Centre for Smoking Cessation and Training reviewed, evaluated and compared 26 national guidelines. Almost all guidelines place importance on identifying smokers, advising them to quit and providing behavioural and medication-based support. However, there were discrepancies in the support offered, which could be due to different interpretations of evidence, costs of medication and cultural differences. The authors offer a checklist for primary care that can inform future universal guidelines suitable for primary care.

## Introduction

Tobacco smoking is a major preventable risk factor for the development of non-communicable diseases, including cancers, cardiovascular and respiratory diseases.^1^ Consequently, 12% of all adult deaths worldwide are attributable to tobacco use.^2^ Overall, among those aged 15 years and over, the worldwide prevalence of tobacco use is 22%. Smoking prevalence is, however, substantially higher among males (36%) than females (8%) (ref. 3), with large variation across countries ranging between 22% (Brazil) and 60.6% (Russia) among males, and between 0.6% (Egypt) and 28.7% (Bangladesh) among females.^3,4^

Long-term smoking cessation substantially reduces health risks^5,6^ and leads to a decrease in the risk of early mortality.^7^ Nationally implemented services for smoking cessation support, such as face-to-face support^8^ and quit lines,^9^ have been found to be effective in helping smokers to quit. Easy access to such smoking cessation treatment and support has also shown to increase quit rates.^10^

In many countries, smokers are most often identified, advised and offered quit support in a primary care setting.^11^ In countries with established specialist cessation services (e.g., face-to-face services and/or quit lines), general practice is the optimum environment for the identification and referral of smokers to take place. For example, in the UK almost 300 million smoking cessation consultations a year and around 90% of all National Health Service contacts take place in a general practice setting.^12^ Evidence for the effectiveness of interventions in this setting is well established^13^; rates of smoking abstinence are increased when health professionals identify smokers, prompt quit attempts,^13^ and provide assistance to quit smoking, including pharmacotherapy.^14,15^

Guidelines in which this evidence is communicated to health professionals can be effective in improving clinical practice,^16^ although the effects depend upon factors such as guideline quality, context and professional experience.^17–19^ Guidelines also offer an opportunity for raising the profile of smoking cessation and facilitate the implementation of the WHO Framework Convention on Tobacco Control.^20^

This study aims to assess the nature and extent of the current national guidelines available for the treatment of tobacco dependence in primary care. As such, the objective of the study is threefold and includes an assessment of: (1) the parties involved in the development of these guidelines, (2) the recommendations made within these guidelines for primary care and (3) how these recommendations correlate with each other (consistency) and with the state-of-the-art evidence of what is effective (validity).

## Results

### Guideline inclusion

**Fig. 1:**
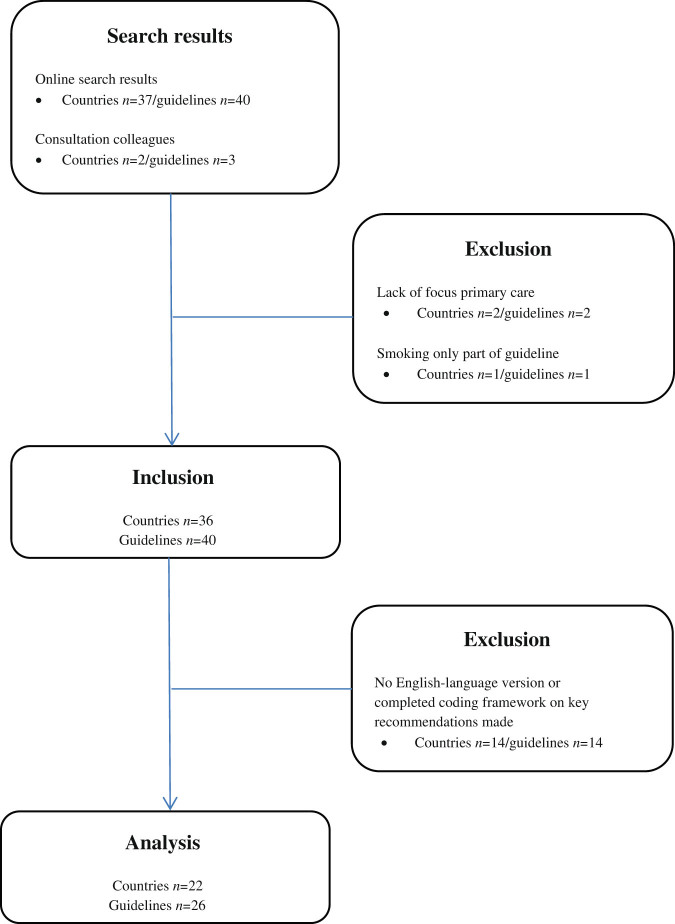
Flowchart of the study

We identified a total of 43 guidelines from 39 countries. After initial review, we excluded three guidelines. Reasons for exclusion were that they either merely presented minimum specifications for a national smoking cessation service (Northern Ireland and Spain), or they focused on treatment of asthma and chronic obstructive pulmonary disease (COPD) other than on smoking cessation (Greece). Of the remaining 40 guidelines, only 16 guidelines were initially available in English. Authors of another 10 guidelines were willing to complete our coding framework on the key recommendations made within the guidelines, resulting in a total of 26 guidelines that were fully assessed for this review (Fig. 1). Table 1 provides a detailed overview of the final inclusion.

**Table 1:** Details of identified national guidelines for smoking cessation in primary care

Country	Income region	Guideline focus	Most recent publication	Lead organisation	Author(s)	Funding source	Development methodology	
Argentina	Upper middle	General	2011	Ministry of Health, National Quality Assurance Program in Health Care	Casetta, B. and Videla, A	None reported	Systematic literature review (guidelines and meta-analysis). Levels of evidence assigned to recommendations. Reviewed by expert panel.	
Australia	High	General	2011	Royal Australian College of General Practitioners	Zwart *et al.*	Private	Not described. Levels of evidence and strength of recommendation categories assigned to recommendations	
Canada	High	General	2011	The Canadian Action Network for the Advancement, Dissemination and Adoption of Practice-informed Tobacco Treatment (CAN-ADAPPT); Centre for Addiction and Mental Health	Selby *et al*.	Drugs and Tobacco Initiative, Health Canada	Review and appraisal of existing English-language clinical practice guidelines and systematic search for evidence. Levels of evidence assigned to recommendations	
		Pregnancy	2010	CAN-ADAPPT; Centre for Addiction and Mental Health	Ordean, A.	Drugs and Tobacco Initiative, Health Canada	Review and appraisal of existing English-language clinical practice guidelines and systematic search for evidence. Evidence levels assigned to each recommendation	
Chile	High	General	2003	Ministry of Health, Pan American Health Organisation	Marisol Acuña	None reported	Not reported	
Czech Republic	High	General	2005	—	Králíková, E.	None reported	Not reported	
Denmark	High	General	2011	Danish Health and Medicines Authority	Pisinger * et al.*	Ministry of Health	Based on thorough review of available guidelines from England, US, Canada, Australia and New Zealand and Cochrane reviews on smoking cessation. Tailored for Danish conditions. Written by tobacco research expert in cooperation with a general practitioner, a representative from the municipalities and a representative from smoking cessation counsellors network	
France	High	General	2007	Health authority	Scemama *et al.*	Public funds	Document review, expert panel	
		Pregnancy	2004	Alliance against tobacco	Delcroix *et al.*	State Insurance Fund for Free-lance Professionals, Nord-Pas-de-Calais Regional Council, Health Protection Branch, National League against Cancer, National Mutual Insurance Company of Hospital Staff, Aventis; EOLYS; FIM; GlaxoSmithKline; Novartis Santé Familiale; Pfizer Pierre Fabre Santé; Roche Nicholas	Presentation of evidence by experts to a jury responsible for drafting the guidelines	
		Peri-operative	2005	French Association of Surgery	Cohendy *et al.*	Ministère de la Santé DGS; Pfizer; AltanaPharma; Sanofi-Aventis; Glaxo SmithKline; Novartis; Pierre Fabre Santé	Literature review	
Germany	High	General	2004	Association of the Scientific Medical Societies in Germany (AWMF)	Batra *et al.*	Donations: DG Sucht and DGPPN, Support: Central Institute of Mental Health, department Addiction Research and Addiction Medicine in Tübingen. No third-parties or private companies	Systematic literature and critical appraisal. Level of evidence assigned to recommendations. Afterwards reviewed by an expert panel	
		COPD	2008	German Society for Pneumology and Respiratory Medicine	Andreas *et al.*	None reported	Literature review and review by expert panel	
India	Lower-middle	General	2011	National Tobacco Control Programme, Directorate General of Health Services, Ministry of Health and Family Welfare, Government of India	Rajkumar *et al.*	None reported	Not described	
Japan	High	General	2010	Japanese Circulation Society	Japanese Circulation Society Joint Working Group	None reported	Not described. Levels of evidence assigned to recommendations	
Jordan	Upper-middle	General	2014	King Hussein Cancer Foundation, King Hussein Cancer Center	Hawari *et al.*	None reported	Literature review	
Kyrgyzstan	Lower-middle	General	2004	Ministry of Healthcare of Kyrgyz Republic	Brikulov *et al.*	None reported	Not described	
Malaysia	Upper-middle	General	2003	Ministry of Health Malaysia	Aziahbt Mahayiddin *et al.*	None reported	Adaptation of US (2000), New Zealand (2001) and American Psychiatric Association (1996) guidelines with incorporation of a systematic literature review. Levels of evidence assigned to recommendations.	
Netherlands	High	General	2007	Dutch Association of General Practitioners	Chavannes *et al.*	Stop Smoking Partnership	Alignment with the multidisciplinary guideline regarding tobacco addiction developed by the Dutch Institite for Healthcare Improvement and adapted for use in general practice (2004)	
New Zealand	High	General	2014	Clinical Trials Research Unit (now: the National Institute for Health Innovation [NIHI]), the University of Auckland	McRobbie *et al.*	Ministry of Health	Literature review undertaken by a consortium. Guidelines developed in accordance with the AGREE tool. Levels of evidence assigned to recommendations according to the New Zealand Guidelines Group.	
Norway	High	General	2004	Health and Social Affairs Agency	Huseby *et al.*	None reported	Not described	
Portugal	High	General	2008	Centre for Evidence Based Medicine, University of Lisbon School of Medicine	Reis *et al.*	Pfizer (unrestricted grant)	Not described	
Scotland	High	General	2004	Health Scotland, Action on Smoking and Health Scotland	West *et al.*	None reported	Not described	
South Africa	Upper-middle	General	2013	South African Thoracic Society	van Zyl-Smit *et al.*	Pfizer	Review and appraisal of existing international clinical practice guidelines, applying them specific national needs. Evidence assigned to each recommendation	
Sweden	High	General	2011	The National Board of Health and Welfare	Axelsen *et al.*	None reported		
UK	High	General	2000	Health Education Authority	West *et al.*	Health Education Authority; Health Development Agency	Based on meta-analytic reviews and other relevant evidence. Levels of evidence assigned to recommendations	
USA	High	General	2008	U.S. Department of Health and Human Services	Fiore *et al.*	Agency for Healthcare Research and Quality; Centers for Disease Control and Prevention; National Cancer Institute; National Heart, Lung, and Blood Institute; National Institute on Drug Abuse; American Legacy Foundation; Robert Wood Johnson Foundation; University of Wisconsin School of Medicine; Public Health’s Center for Tobacco Research and Intervention	Systematic literature review. Levels of evidence assigned to recommendations	

### Parties involved in guideline development

Most guidelines were produced/commissioned by governmental organisations, followed by medical societies, multiple organisations, research networks and research centers. General practitioners (GPs) were involved in the development of the majority of the included guidelines (19/26); in six guidelines the lead-author of the guideline was a GP. In three guidelines, the development was led by an association of GPs and in ten guidelines at least one GP was involved in the development among many other authors. Only four guidelines were developed without any involvements of GPs and for the remaining three guidelines it was not possible to identify the profession of the authors and, therefore, GP involvement remains unclear (Japan, India, South Africa).

### Guideline recommendations for primary care

The majority of the included guidelines focused on smoking cessation in the general population (*n* = 22), two focused specifically on smoking cessation during pregnancy (France and Canada), one on smoking cessation among COPD patients (Germany) and one on smoking cessation among perioperative patients (France). Table 1 presents brief details on the funding sources and methodologies used for the development of these guidelines. A list of references for each of the guidelines can be found in Appendix A.

#### General population

With regard to guidelines that focus on the treatment of tobacco dependence in the general population (*n* = 22), we were able to categorise recommendations into 12 intervention types recommended for primary care:

- Each patient’s smoking status should be identified and recorded (20/22);
- All smokers should be given brief advice to stop (20/22);
- Smokers’ motivation to quit should be assessed (17/22);
- All smokers wishing to stop should be offered assistance (22/22);
- All smokers wishing to stop should be offered/encouraged to use pharmacotherapy (22/22);
- All smokers wishing to stop should be offered behavioural support (19/22);
- Self-help materials should be offered as part of tobacco dependence treatment (12/22);
- Smoking abstinence should be evaluated following treatment (5/22);
- The ‘5A’s Framework’ should be used to guide brief intervention (16/22);
- The ‘ABC Framework’ should be used to guide brief intervention (3/22);
- Health-care professionals should be trained in delivering tobacco dependence treatment (13/22);
- Hypnotherapy and acupuncture are not effective smoking cessation treatments (6/22).

Table 2 provides an overview of these recommendations put forth in each national guideline. Full details on the types of pharmacotherapy recommended in each national guideline are presented in Table 3.

**Table 2:** Recommendations in national guidelines for smoking cessation in primary care

	Argentina	Australia	Canada	Chile	Czech Republic	Denmark	France	Germany	India	Japan	Jordan	Kyrgyzstan	Malaysia	Netherlands	New Zealand	Norway	Portugal	Scotland	South Africa	Sweden	UK	USA
Identify	✓	✓	✓	✓	✓	✓	✓	✓	✓	✓	✓	✓	✓		✓	✓		✓	✓	✓	✓	✓
Brief advice	✓	✓	✓	✓	✓	✓		✓	✓	✓	✓		✓	✓	✓	✓	✓	✓	✓	✓	✓	✓
Assess motivation	✓	✓	✓	✓	✓	✓	✓		✓	✓	✓	✓	✓	✓		✓			✓	✓		✓
Offer assistance	✓	✓	✓	✓	✓	✓	✓	✓	✓	✓	✓	✓	✓	✓	✓	✓	✓	✓	✓	✓	✓	✓
Offer pharmacotherapy	✓	✓	✓	✓	✓	✓	✓	✓	✓	✓	✓	✓	✓	✓	✓	✓	✓	✓	✓	✓	✓	✓
Offer behavioural support	✓	✓	✓	✓	✓	✓	✓		✓	✓	✓	✓	✓	✓	✓	✓	✓		✓	✓		✓
Self-help	✓			✓		✓	✓	✓		✓	✓				✓	✓	✓			✓		✓
Evaluate abstinence				✓	✓		✓				✓											✓
5A’s Framework	✓	✓	✓	✓	✓	✓			✓	✓	✓		✓	✓		✓	✓		✓		✓	✓
ABC Framework					✓	✓									✓							
Appropriate training	✓				✓	✓	✓	✓			✓			✓	✓			✓		✓	✓	✓
Hypnotherapy and acupuncture not effective	✓	✓						✓^a^				✓							✓	✓		

Note: 5A’s = Ask, Advise, Assess, Assist, Arrange, *ABC* Ask, provide Brief advice, offer/refer to/provide evidence-based Counselling.

Excluding guidelines targeting specific population

^a^The German guideline recommends hypnotherapy; acupuncture is not recommended as an effective treatment

**Table 3:** Types of pharmacotherapy recommended in national guidelines for smoking cessation in primary care

	Argentina	Australia	Canada^a^	Chile	Czech Republic	Denmark	France	Germany	India	Japan	Jordan	Kyrgyzstan	Malaysia	Netherlands	New Zealand	Norway	Portugal	Scotland^b^	South Africa	Sweden	UK	USA
NRT	✓	✓		✓	✓	✓	✓	✓	✓	✓	✓	✓	✓	✓	✓	✓	✓		✓	✓	✓	✓
Bupropion	✓	✓		✓	✓	✓	✓	✓	✓	✓		✓	✓	✓	✓	✓	✓			✓	✓	✓
Varenicline	✓	✓			✓	✓	✓	✓	✓	✓			✓		✓		✓		✓	✓	✓	✓
Combination NRT	✓	✓		✓	✓	✓	✓	✓		✓	✓	✓	✓	✓	✓	✓	✓		✓	✓	✓	✓
Combined NRT + Bupropion	✓			✓	✓			✓			✓		✓	✓					✓			✓
Nortriptyline	✓^c^	✓^c^						✓			✓^c^			✓	✓		✓		✓			✓^c^
Clonidine	✓^c^										✓^c^						✓^c^					✓^c^

Note: *NRT* nicotine replacement therapy.

Excluding guidelines targeting specific populations

^a^The Canadian guideline did not include a section of recommendations regarding pharmacotherapy. It was noted within these guideline that the development of these recommendations was in progress. At the time of writing this paper an update including recommendations for pharmacotherapy had not been released

^b^The Scottish guidelines did include recommendations for pharmacotherapy, however, these were included within recommendations for Specialist Stop Smoking Services which were not extracted for the purpose of this review. These recommended NRT, combination NRT and bupropion

^c^Second line

#### Subpopulations

The majority of the guidelines include sections concerning the treatment of tobacco dependence among specific subpopulations (e.g., pregnant women, children and adolescents, COPD patients). Moreover, several countries developed specific guidelines for the treatment of tobacco dependence among such subpopulations. The most frequently mentioned recommendations across guidelines are described in this section (details on recommendations for specific subpopulations in each national guideline are presented in Table 4).

**Table 4:** Evidence ratings assigned to recommendations from included guidelines

	S&D Guidance (Y/N)	Evidence rating^a^	Argentina	Australia	Canada^b^	Canada pregnancy	Chile	Czech Republic	Denmark	France	France pregnancy	France perioperative	Germany	Germany COPD	India	Japan	Jordan	Kyrgyzstan	Malaysia	Netherlands	New Zealand	Norway	Portugal	Scotland^c^	South Africa	Sweden	UK	USA
General population																												
Identify	N	–	✓	✓	✓		✓	✓	✓	✓			✓		✓	✓	✓	✓	✓		✓	✓		✓	✓	✓	✓	✓
Brief advice	Y	A	✓	✓	✓		✓	✓	✓				✓		✓	✓	✓		✓	✓	✓	✓	✓	✓	✓	✓	✓	✓
Assess motivation	N	—	✓	✓	✓		✓	✓	✓	✓					✓	✓	✓	✓	✓	✓		✓			✓	✓		✓
Offer assistance	N	—	✓	✓	✓		✓	✓	✓	✓			✓		✓	✓	✓	✓	✓	✓	✓	✓	✓	✓	✓	✓	✓	✓
Offer pharmacotherapy	Y	—	✓	✓	✓		✓	✓	✓	✓			✓		✓	✓	✓	✓	✓	✓	✓	✓	✓	✓	✓	✓	✓	✓
NRT	Y	A	✓	✓			✓	✓	✓	✓			✓		✓	✓	✓	✓	✓	✓	✓	✓	✓		✓	✓	✓	✓
Bupropion	Y	A	✓	✓			✓	✓	✓	✓			✓		✓			✓	✓	✓	✓	✓	✓			✓	✓	✓
Varenicline	Y	A	✓	✓				✓	✓	✓			✓		✓	✓					✓		✓		✓	✓		✓
Combination NRT	Y	A	✓	✓			✓	✓	✓	✓			✓			✓	✓	✓	✓		✓	✓	✓		✓	✓	✓	✓
Combined NRT *+* bupropion	N	—	✓				✓	✓					✓				✓		✓	✓					✓			✓
Nortriptyline	N	—	✓^e^	✓									✓				✓^e^			✓	✓		✓		✓			✓
Clonidine	N	—	✓^e^										✓				✓^e^						✓					✓
Offer behavioural support	Y	A	✓	✓	✓		✓	✓	✓	✓			✓		✓	✓	✓	✓	✓	✓	✓	✓	✓		✓	✓		✓
Self-help	N	—	✓				✓		✓	✓			✓			✓	✓			✓	✓	✓	✓			✓		✓
Evaluate abstinence	Y	A					✓	✓		✓							✓											✓
5A’s framework	N	—	✓	✓	✓		✓	✓	✓						✓	✓	✓		✓	✓		✓	✓		✓		✓	✓
ABC framework	N	—						✓	✓												✓							
Appropriate training	N	—	✓					✓	✓	✓			✓				✓			✓	✓			✓		✓	✓	✓
Subpopulations																												
Pregnancy	Y	B	✓	✓		✓	✓	✓		✓	✓		✓		✓	✓		✓	✓	✓	✓	✓	✓	✓	✓	✓	✓	✓
Offer NRT	Y	C	✓	✓		✓	✓	✓		✓	✓		✓		✓				✓	✓	✓		✓		✓	✓	✓	
BME groups	Y	B			✓																✓	✓	✓			✓		
Children and adolescents	Y	—	✓		✓		✓	✓		✓	✓		✓			✓	✓		✓	✓	✓	✓	✓			✓		✓
Offer NRT	N	—	✓				✓	✓		✓^d^			✓			✓	✓		✓	✓	✓					✓		
Mental illness/other addiction	Y	B/C	✓		✓			✓		✓			✓		✓				✓		✓		✓		✓	✓		
Bupropion	N	—	✓					✓		✓			✓						✓				✓					
Nortriptyline	N	—											✓										✓					
Chronic somatic illness	N	—	✓	✓				✓		✓			✓	✓	✓	✓	✓	✓		✓	✓		✓			✓	✓	
Offer NRT (CVD)	N	—	✓	✓			✓	✓		✓			✓		✓	✓	✓			✓	✓		✓				✓	
Bupropion (CVD)	N	—	✓				✓	✓							✓					✓	✓		✓					

Note: *S&D* Service and Delivery Guidance, *NRT* nicotine replacement therapy, *CVD* cardiovascular disease, *COPD* chronic obstructive pulmonary disease, *BME* black and minority ethnic groups

^a^Evidence rating based on the SIGN system: A=recommendation is supported by strong evidence, B=recommendation is supported by reasonable evidence, C=recommendation is supported by expert opinion only, I=insufficient evidence to make a recommendation

^b^The Canadian guideline did not include a section of recommendations regarding pharmacotherapy. It was noted within these guideline that the development of these recommendations was in progress. At the time of writing this review an update including recommendations for pharmacotherapy had not been released

^c^The Scottish guideline did include recommendations for pharmacotherapy, however, these were included within recommendations for Specialist Stop Smoking Services which were not extracted for the purpose of this review. These recommended NRT, combination NRT and bupropion

^d^The French guideline for the general population recommends NRT only for adolescent of 15 years and older

^e^Second line

#### Pregnant women

In total, 19 guidelines targeting the general population provided recommendations for the treatment of tobacco dependence among pregnant women. In addition, France and Canada developed a specific guideline for this subpopulation. Overall, guidelines recommend that all pregnant smokers should be offered brief advice to quit and should be provided with counselling, including behavioural and pharmacological support. Of the 21 guidelines that address smoking cessation treatment among pregnant women, 16 guidelines recommended that nicotine replacement therapy (NRT) can be used to assist smoking cessation attempts made by pregnant women. In several countries, intermittent-dosage forms of NRT (e.g., gum, nasal and oral sprays) are recommended as a preferred pharmacotherapy over patches (Australia, Canada (pregnancy-specific guideline), New Zealand, Portugal and India). In contrast, guidelines from Norway, Scotland, the United States, Japan and Kyrgyzstan all recommended that NRT should not be given to pregnant women.

#### Black and minority ethnic groups

Guidelines from five countries made recommendations for black and minority ethnic (BME) groups: Canada, New Zealand, Norway, Portugal and Sweden. All recommend that wherever possible, culturally appropriate smoking cessation support should be offered. These guidelines also recommend that health-care workers should receive additional training in delivering smoking cessation support to BME groups.

#### Children and adolescents

In total, 16 guidelines made specific recommendations for children and adolescents. Overall, these include: (1) information about tobacco use among children and adolescents should be obtained on a regular basis, (2) children and adolescents should be counselled to encourage abstinence and (3) parents who smoke should be offered smoking cessation support in order to limit children's exposure to secondhand tobacco smoke. Of these guidelines, 11 guidelines recommend that NRT could be offered to adolescents who show evidence of nicotine dependence. In contrast, several other guidelines recommend that pharmacotherapy should not be offered to children and adolescents (Canada, Norway, Portugal and the USA).

#### Mental illness/other addiction

Eleven guidelines made specific recommendations for smokers with a mental illness or other substance addiction. Most commonly, guidelines recommend to offer counselling that incorporates all known effective components and to carefully monitor smoking cessation in which medication dosages should be adjusted if necessary. The types of pharmacotherapy recommended for the treatment of tobacco dependence among these patients differed across countries (Table 4).

#### Chronic illness

In total, 15 guidelines made specific recommendations for people with a chronic illness. Most guidelines recommend that NRT is safe to use in people with stable cardiovascular disease. However, NRT should be used with caution in patients with unstable cardiovascular disease (e.g., severe or unstable angina, recent myocardial infarction). Additionally, several guidelines also recommend bupropion as a safe treatment for nicotine dependence in people with cardiovascular disease (Argentina, Chile, Czech Republic, India, the Netherlands, New Zealand and Portugal).

### Validity of guideline recommendations

Table 4 lists all recommendations made in the included guidelines and shows the level of evidence we assigned to them according to the Scottish Intercollegiate Guidelines Network (SIGN) system. Of all recommendations, only three (‘provide brief advice’, ‘provide behavioural support’, and ‘offer/encourage pharmacotherapy’) were included in the Service and Delivery Guidance and as such were assigned an evidence rating. We assigned the evidence rating ‘A’ (strong evidence) to ‘provide brief advice’ and ‘provide behavioural support’. With some exceptions, the majority of the included guidelines recommend the use of NRT, a combination of several forms of NRT, bupropion and varenicline; all of which we assigned the evidence rating ‘A’. Fewer guidelines recommended combined treatment with NRT and bupropion, or the use of nortryptiline or clonidine as second-line treatments. These recommendations were not included in the Service and Delivery guidance and as such have not been assigned an evidence rating.

Recommendations concerning pregnant women, BME groups and people with mental illness were assigned an evidence rating ‘B’ (supported by reasonable evidence). People with other addictions were assigned the rating ‘C’ (supported by expert opinion only). For the purposes of our review, mental illness and other substance addictions were placed in a single group to reflect the content of the national guidelines; these groups are treated separately in the Service and Delivery Guidance. Children and adolescents were not assigned an evidence rating as a priority group. Rather, the Service and Delivery Guidance assigned a rating of ‘I’ (insufficient evidence to make a recommendation) to stop smoking interventions and ‘B’ to prevention and tobacco control. The use of NRT for pregnant women was assigned the evidence rating ‘C’. The use of pharmacotherapy for children and adolescents, people with cardiovascular disease and people with mental illness were not assigned an evidence rating as these were not included in the Service and Delivery Guidance.

## Discussion

This study aimed to assess: (1) the parties which were involved in the development of national guidelines for smoking cessation, (2) the recommendations that are made for primary care within these guidelines and (3) how these recommendations correlate with each other (consistency) and with the state-of-the arts evidence of what is effective (validity).

### Main findings

In our study, 26 guidelines from 22 countries were included in the analyses. Four of these guidelines focused on the treatment of tobacco dependence among specific subpopulations: pregnant smokers (Canada and France), perioperative (France) and COPD patients (Germany). Most guidelines were produced/commissioned by governmental organisations, followed by medical societies, multiple organisations, research networks and research centers. Although most guidelines were developed in collaboration with GPs, only a minority of the guidelines was developed by a GP as the lead author.

Overall, recommendations that focused on the treatment of tobacco dependence in primary care among the general population corresponded well with each other across guidelines. The majority of these guidelines recommended that smokers should be identified, be offered a brief advice to quit, be assessed for motivation to quit, and be offered assistance to quit with behavioural support and pharmacotherapy. Also, the majority recommended the use of the ‘5A’s’ framework to guide brief intervention.

We found more inconsistency across details of specific recommendations. For example, the specific content (practical counselling techniques vs. no details on content) and delivery format (e.g., only telephone or face-to-face vs. multiple formats) of behavioural support differed greatly throughout guidelines. These inconsistencies can be partly explained by the differences in the services available at the country level (e.g., the Australian guideline mentioned telephone support since they have a national quitline).

Other inconsistencies among recommendations were related to the provision of pharmacotherapy for smoking cessation among the general population. Excluding the Canadian and Scottish guidelines, who did not make recommendations relating to the use of pharmacotherapy, NRT was recommended by all guidelines. Although a combination of NRT products was recommended by a majority of the guidelines (19/22), varenicline was recommended by only 15 of 22 guidelines. Recommendations to the use of nortriptyline (9/22), a combination of NRT and bupropion (9/22), and clonidine (4/22) were less common. The discrepancies between these recommendations may be due to differences in medication licensing across countries, or to a difference in access or interpretation of the current available scientific evidence. Also, we argue that costs of medication could be a possible barrier in certain countries for uptake of certain recommendations into the guideline.

Finally, recommendations for specific subpopulations were also less consistent throughout guidelines. For example, 15 guidelines recommended that NRT could be used when needed for pregnant women, while five guidelines recommended that NRT should not be used during pregnancy. Similarly, 10 guidelines recommended that NRT could be used for children and adolescents, while four recommended that NRT should not be used in this group. Possibly these inconsistencies are related to the level of evidence available for these subpopulations, which mostly undefined and otherwise limited to level B or lower (Table 4). Cultural infuences—such as the position of pharmacotherapy as well as the position of the subpopulations in society—may play a role as well.

### Strengths and limitations of this study

The current study is one of the first to systematically identify and analyse the nature and extent of a large number of practice guidelines for the treatment of tobacco dependence in primary care. Moreover, it is the first to compare guideline recommendations across countries and with state-of-the-art evidence for both the general population and specific subpopulations.

However, some limitations need to be taken into account when interpreting the results. Firstly, we only included guidelines that were written or translated into English. Non-English guidelines that could not be translated were only included if the guideline authors were willing to provide the necessary information, and data was thus not derived directly from these guidelines.

Secondly, several guideline recommendations made in the Service and Delivery Guidance were not mentioned in any of the included national guidelines. These include types of pharmacotherapy treatment (nicotine-assisted reduction to stop—evidence rating B), unlicensed stop smoking treatment (e.g., electronic cigarettes—evidence rating C), and the identification of routine and manual workers as an additional priority group (evidence rating B). The lack of uptake of recommendations regarding electronic cigarettes can be explained by their recent rapid rise on the market, which mainly started only after the development of most of the included guidelines (most of them dating from 2011 and earlier). It illustrates a need of frequent updates of the national guidelines. The lack of inclusion of other recommendations in the identified guidelines may indicate a translational gap of the latest evidence. We, therefore, recommend future studies that aim to identify this translational gap, raise awareness among guideline developers and trigger them to update guidelines and include the latest evidence.

Thirdly, we were unable to rate the quality of several recommendations within the guidelines because they did not match with the recommendations within the Service and Delivery Guidance (identify smokers’, ‘assess motivation’, ‘offer assistance’ and ‘use the 5 A’s framework’). This was likely due to vague descriptions of the recommendations within the guidelines.

Finally, although we managed to analyse the consistency and validity of the guideline recommendations, this study does not provide insight into how these guideline recommendations are currently being implemented in each country.

### Implications for future research and practice

Results of our study indicate that, although some consensus on smoking cessation recommendations already exist, there is room for improvement with regard to the inconsistencies we found. Therefore, we suggest that the development of an international smoking cessation guideline for primary care, drawing upon the latest evidence and written by international clinical, policy and academic experts could provide a template to optimise future national guidelines. Based on our study findings, we developed a checklist of recommended smoking cessation intervention components as a ‘tool for practice’ for primary care professionals (Supplementary Material). Although this checklist presents universal key recommendations for the treatment of tobacco dependence in primary care, both health professionals and guideline developers need to take into consideration their own national health- and economic context when applying these recommendations. Among these considerations should be cultural adaptations tailored to, for example, specific risk groups.

## Conclusions

Our study shows that there is almost universal agreement between guidelines regarding the need in primary care to identify smokers, to offer some form of advice to quit and to offer behavioural and pharmacological support to quit. Discrepancies between guideline recommendations were greatest for these latter interventions, which are likely due to different interpretations of the evidence and/or differences in the contextual health environments of countries. International primary care guidance for smoking cessation that is dynamic, (co-) written by primary care experts and drawing from the latest evidence would be a very useful resource for clinicians and policy makers to develop and optimise national guidelines.

## Methods

### Search methods and inclusion criteria

To identify national guidelines for smoking cessation, we used an independent online resource which hosts a compilation of national treatment guidelines from across the world (www.treatobacco.net). Additional search methods included the consultation of international colleagues within the field of tobacco dependence treatment. The initial search was performed in December 2014 and was updated in September 2015. Only the most recent version of each available national guideline was included. If multiple guidelines for specific subpopulations (e.g., pregnant women) were available for one country, all those guidelines were included.

Only English-language guidelines were eligible for analyses. If guidelines were not available in English, we contacted the lead authors of the guideline and asked if they could provide an English-language version. If unsuccessful, we then asked if they were willing to provide details on the key recommendations made within the guidelines (section ‘Guideline recommendations’). In case the communication with an author resulted in the availability of a more recent version of the guideline or to a version with a more specific focus on primary care, this version of the guideline replaced the initially included guidelines of this country.

We excluded guidelines where smoking cessation was only part of a guideline on a different topic (e.g., a paragraph on tobacco treatment within a guideline for asthma or COPD).

### Data extraction

From each country’s guideline we extracted information regarding the lead authors, the year of publication, and the lead organisation(s) involved in commissioning and/or producing the guideline (e.g., Ministry of Health). We also extracted information on funding sources for guideline development and on the development methodology. We classified the country as a high-, middle- or low-income country based on the information from the World Bank income group (http://data.worldbank.org/about/country-and-lending-groups).

### Guideline recommendations

In order to identify the recommendations made, we extracted the main recommendations put forth in each country's guideline. For each included guideline, we created a coding framework consisting of the recommendations within the guideline. A segment of text was extracted as a recommendation if it met the following criteria: (1) the text was explicitly specified as a recommendation and (2) the recommendation was made specifically for a primary care setting or primary healthcare practitioner, or was relevant to either one of these. Recommendations were extracted in their smallest, irreducible form. For example, a recommendation might state that “pregnant women should only be offered NRT if they are unable to quit otherwise, and intermittent forms of NRT are preferable to patches in this population”. We recorded this as two separate recommendations: (1) “pregnant women should only be offered NRT if they are unable to quit otherwise” and (2) “intermittent forms of NRT are preferable to patches for pregnant women”. Recording the recommendations in this way facilitated the comparison of recommendations across guideline documents.

To ensure consistency in the extraction of recommendations, two researchers (K.S. and G.A.) independently extracted the recommendations from two pre-selected guidelines. These two researchers discussed any discrepancies in their extraction and resolved them through discussion or through arbitration with the third researcher (A.Mc.). After having agreed upon a consistent approach to extraction, K.S. and G.A. extracted both 50% of the remainder guideline recommendations.

### Consistency of guideline recommendations

To establish how consistent the recommendations were internationally, we compared each of the individual national recommendations and contrasted them against all others. Those recommendations that were identical, or very similar, were assigned to categories (e.g., ‘give all smokers brief advice to quit’ or ‘record and update tobacco use status for all patients’). We used a ‘pile-sort’ method in order to establish this categorisation. Each individual recommendation was printed out on a separate piece of card along with a unique identifier. The cards were sorted independently by two researchers (K.S. and G.A.) into piles of identical/very similar individual recommendations. Each researcher assigned a category label to individual piles, and subsequently met to discuss the categorisations. Disagreements in categorisation were discussed with the third author (A.Mc.) until resolved, with category labels being finalised and agreed upon at this stage.

### Validity of guidelines recommendations

To establish how well the recommendations in each of the guidelines correspond to state-of-the-art evidence, recommendations were cross-checked against the most recent English Service and Delivery Guidance for local Stop Smoking Services.^21^ This publication makes recommendations for how stop smoking services in the UK should be commissioned, delivered and monitored. Most importantly, it includes an updated evidence review of a number of recommendations for the treatment of tobacco dependence, including behavioural support, pharmacotherapy and the treatment among specific subpopulations.

Each recommendation within the included national guidelines was assigned an evidence rating using the SIGN rating system.^22^ The SIGN system includes five ratings: “A” (the recommendation is supported by good/strong evidence), “B” (the recommendation is supported by fair/reasonable evidence, but there may be minimal inconsistency or uncertainty), “C” (the recommendation is supported by published expert opinion only), “I” (there is insufficient evidence to make a recommendation) and “✓” (good practice point in the opinion of the guidance development group).

### Checklist of smoking cessation intervention components

We developed a checklist of components as a practice tool for primary care professionals based on: (1) the analysis of recommendations in the identified national guidelines, (2) the consistency and validity of these recommendations and (3) experts’ opinions from the International Primary Care Respiratory Group. 

## Electronic supplementary material

Supplementary Appendix

Supplementary Material
