# Supplementary material for: National guidelines for smoking cessation in primary care: a literature review and evidence analysis
Source: NPJ Prim Care Respir Med. 2017 Jan 20;27:2. doi: 10.1038/s41533-016-0004-8 (PMC5434788; doi:10.1038/s41533-016-0004-8)
Supplement: Supplementary file 1 — Supplementary Appendix [file 41533_2016_4_MOESM1_ESM.docx]

**Appendix A. Guideline references**

**Argentina**

Ministerio de Salud. *Guía de Práctica Clínica Nacional de Tratamiento de la Adicción al Tabaco: Recomendaciones basades en la evidencia científica.* Argentina: Ministerio de Salud (2011).

**Australia**

Zwar N, Richmond R, Borland R, Peters M, Litt J, Bell J et al. *Supporting smoking cessation: a guide for health professionals.* Melbourne: Royal Australian College of General Practitioners (2011).

**Canada – general population**

CAN-ADAPPT. *Canadian Smoking Cessation Clinical Practice Guideline*. Toronto, Canada: Canadian Action Network for the Advancement, Dissemination and Adoption of Practice-informed Tobacco Treatment; Centre for Addiction and Mental Health (2011)

**Canada – pregnancy-specific**

CAN-ADAPPT. *Canadian Smoking Cessation Guideline: Specific Populations: Pregnant and Breastfeeding Women*. Toronto, Canada: Canadian Action Network for the Advancement, Dissemination and Adoption of Practice-informed Tobacco Treatment; Centre for Addiction and Mental Health (2011)..

**Chile**

Acuña M. *La cesación del consumo de tabaco: manualPara el equipo de salud.* Santiago, Chile: Ministerio de Salud; Pan American Health Organisation (2003)

**Czech Republic**

Králíková E, Býma S, Cífková R, Češka R, Dvořák V, Hamanová J et al. Doporučení pro léčbu závislosti na tabáku. *Časopis Lékařů Českých*, **144**: 327-333 (2005).

**Denmark**

Pisinger CH. *Behandling af tobaksafhængighed – Anbefalinger til en styrket klinisk praksis.* København, Danmark: Sundhedsstyrelsen (2011).

**Finland**

Winell K, Jousilahti P, Kauppi P, Korhonen T, Ollila H, Pietalä K et al. *Tupakkariippuvuus ja tupakasta vieroitus*. Helsinki, Finland: Käypä Hoito (2012).

**France – general population**

Haute Autorité de Santé. *Strategies therapeutiques d’aide au sevrage tabagique: Efficacité, efficience et prise en charge financière*. Paris, France: Haute Autorité de Santé (2007).

**France – preganacy-specific**

ANAES. *Consensus conference: Pregnancy and tobacco.* Paris, France: Agence nationale d’accréditation et d’évaluation en santé (2004).

**France – perioperative-specific**

French Conference of Experts: Perioperative smoking control. Reference cannot be given as guidelines could not be translated (2005).

**Germany – general population**

*Tabakbedingte Störungen: “Leitlinie Tabakentwöhnung*. Germany: Arbeitsgemeinschaft der Wissenschaftlichen Medizinischen Fachgesellschaften Leitlinien (2004).

**Germany – COPD-specific**

Andreas S, Batra A, Behr J. Berck H, Chenot J-F, Gillissen A et al. Tabakentwöhnung bei COPD: S3 Leitlinie herausgegeben von der Deutschen Gesellschaft für Pneumologie und Beatmungsmedizin. *Pneumologie*, **62**: 255-272 (2008).

**India**

National Tobacco Control Programme. *Tobacco Dependence Treatment Guidelines*. New Delhi, India: Directorate General of Health Services, Ministry of Health & Family Welfare, Government of India (2011).

**Japan**

Japanese Circulation Society Joint Working Group. *Circulation Journal*, **76**: 1024-1043 (2012).

**Jordan**

Hawari F, Ayub H. Obeidat N. & Habashneh M. *Jordan guidelines for tobaccodependence treatment “Helping smokers quit”*. Jordan: King Hussein Cancer Society (2014).

**Kyrgyzstan**

Brimkulov NN,Vinnikov DV & Cholurova RA. *Guidelines on Tobacco Dependence Treatment*. Bishkek, Kyrgyz Republic: Kyrgyz Asthma Centre (2004).

**Malaysia**

Ministry of Health. *Clinical Practice Guidelines on Treatment of Tobacco Use and Dependence*. Malaysia: Ministry of Health (2003)

**Netherlands**

Chavannes NH, Kaper J, Frijling BD, Van der Laan JR, Jansen PWM, Guerrouj S et al. NHG-Standaard Stoppen met roken. *Huisarts Wet*, **50** :306-14 (2007).

**New Zealand**

Ministry of Health. 2014. The New Zealand Guidelines for Helping People to Stop Smoking. Wellington: Ministry of Health.

**Norway**

Sosial- og helsedirektoratet. *Røykeavvenning i primærhelsetjenesten: Retningslinjer for primærhelsetjenestens arbeid med røykeavvenning.* Oslo, Norway: Sosial- og helsedirektoratet (2004).

**Portugal**

Reis I, Fortuna P, Ascenção R, Costa J, Bugalho A. & Vaz Carneiro A. *Clinical Pracic Guideline on Smoking Cessation*. Lisbon, Portugal: Centre for Evidnce Based Medicine (2008).

**Scotland**

West R, McNeill A & Raw M. *Smoking Cessation Guidelines for Scotland*. Edinburgh, Scotland: Health Scotland (2004).

**South Africa**

Van Zuyl-Smit RN, Allwood B, Stickells D, Symons G, Abdool-Gaffar S, Murphy K, Lalloo U, Vanker A, Dheda K & Richard G. South African tobacco smoking cessation clinical practice guideline. *South Afr Med J*, **103**: 869-876 (2013).

**Sweden**

Socialstyrensen. *Nationella riktlinjer för sjukdomsförebyggande metoder 2011: Tobaksbruk, riskbruk av alkohol, otillräcklig fysisk aktivitet och ohälsosamma matvanor.* Sweden: Socialstyrensen (2011).

**UK**

West R, McNeill A & Raw M. Smoking cessation guidelines for health professionals: an update. *Thorax*, **55**: 987-999 (2000).

**USA**

Fiore MC, Roberto Jaén C, Baker TB, Bailey WC, Benowitz NL, Curry SJ et al. *Treating Tobacco Use and Dependence: 2008 Update.* USA: U.S. Department of Health and Human Services (2008).
