# Supplementary material for: National guidelines for smoking cessation in primary care: a literature review and evidence analysis
Source: NPJ Prim Care Respir Med. 2017 Jan 20;27:2. doi: 10.1038/s41533-016-0004-8 (PMC5434788; doi:10.1038/s41533-016-0004-8)
Supplement: Supplementary file 2 — Supplementary Material [file 41533_2016_4_MOESM2_ESM.docx]

**Supplementary material: Checklist of components to be included in future primary care guidelines for smoking cessation**

The following checklist suggests intervention components that should be included in any future primary guidelines for smoking cessation, drawn from the findings of this study and in consultation with experts from the International Primary Care Respiratory Group.

| **Intervention** | **Evidence rating*** |
| --- | --- |
| **Provide brief advice** |  |
| Establish and record smoking status (ASK) | I |
| Advise on the harms of smoking and benefits of quitting (ADVISE) | A |
| Provide support to quit (ACT) | I |
| **Provide assistance to quit** |  |
| Assess motivation to quit quantitatively | A |
| Offer pharmacotherapy | I |
| Nicotine replacement therapy (NRT) | A |
| Combination NRT | A |
| Nicotine-Assisted Reduction to Stop (NARS) / preloading | B |
| Unlicensed nicotine containing products (nicotine vapourisers) | C |
| Bupropion (Zyban®) | A |
| Varenicline (Champix®) | A |
| Offer behavioural support | A |
| One-to-one support | A |
| Couple/family support | I |
| Closed-group support | A |
| Open (rolling) group support | B |
| Drop-in support | I |
| Telephone support (proactive) | A |
| Telephone support (reactive) | B |
| Telephone support (text-based) | B |
| Online support | B |
| Offer referral to specialist smoking cessation services | I |
| Offer referral to quit line | I |
| Offer self-help materials | I |
| Evaluate abstinence using biochemical verification | A |

*Evidence rating based on the SIGN system: A=recommendation is supported by strong evidence, B=recommendation is supported by reasonable evidence, C=recommendation is supported by expert opinion only, I=insufficient evidence to make a recommendation
